# Explicit-Duration Hidden Markov Model Inference of UP-DOWN States from Continuous Signals

**DOI:** 10.1371/journal.pone.0021606

**Published:** 2011-06-28

**Authors:** James M. McFarland, Thomas T. G. Hahn, Mayank R. Mehta

**Affiliations:** 1 Department of Physics, Brown University, Providence, Rhode Island, United States of America; 2 Department of Physics and Astronomy, and Integrative Center for Learning and Memory, University of California Los Angeles, Los Angeles, California, United States of America; 3 Department of Psychiatry, Central Institute for Mental Health, Mannheim, Germany; 4 Behavioural Neurophysiology, Max Planck Institute for Medical Research, Heidelberg, Germany; 5 Departments of Neurology and Neurobiology, University of California Los Angeles, Los Angeles, California, United States of America; Universita' del Piemonte Orientale, Italy

## Abstract

Neocortical neurons show UP-DOWN state (UDS) oscillations under a variety of conditions. These UDS have been extensively studied because of the insight they can yield into the functioning of cortical networks, and their proposed role in putative memory formation. A key element in these studies is determining the precise duration and timing of the UDS. These states are typically determined from the membrane potential of one or a small number of cells, which is often not sufficient to reliably estimate the state of an ensemble of neocortical neurons. The local field potential (LFP) provides an attractive method for determining the state of a patch of cortex with high spatio-temporal resolution; however current methods for inferring UDS from LFP signals lack the robustness and flexibility to be applicable when UDS properties may vary substantially within and across experiments. Here we present an explicit-duration hidden Markov model (EDHMM) framework that is sufficiently general to allow statistically principled inference of UDS from different types of signals (membrane potential, LFP, EEG), combinations of signals (e.g., multichannel LFP recordings) and signal features over long recordings where substantial non-stationarities are present. Using cortical LFPs recorded from urethane-anesthetized mice, we demonstrate that the proposed method allows robust inference of UDS. To illustrate the flexibility of the algorithm we show that it performs well on EEG recordings as well. We then validate these results using simultaneous recordings of the LFP and membrane potential (MP) of nearby cortical neurons, showing that our method offers significant improvements over standard methods. These results could be useful for determining functional connectivity of different brain regions, as well as understanding network dynamics.

## Introduction

During slow-wave sleep, large amplitude slow (<2 Hz) oscillations are present in the EEG and cortical local field potential (LFP) reflecting synchronous fluctuations in the membrane potential (MP) and spiking activity of individual neurons. A similar state is also observed under various anesthetics where neural activity exhibits bistability and undergoes synchronous transitions between a depolarized and active UP state, and a quiescent, hyperpolarized DOWN state [1], [2]. Both excitatory and inhibitory neurons participate in these synchronous state transitions, and thus the active state is characterized by a balance of excitatory and inhibitory activity [3], [4], [5].

UP-DOWN states (UDS) present an excellent opportunity to study both cellular and network properties, and because of their global nature they can yield insight into network dynamics, both within and across brain regions. There has also been much interest in using the relatively simple discrete state dynamics of UDS to study the state-dependence of neural responses to external stimuli [6], [7], [8], [9]. Further, a number of studies have demonstrated that UDS occurring during natural sleep could serve an important role in the process of memory consolidation [10], [11]. This possibility is supported by the observations that hippocampal activity can be synchronized with cortical UDS [12], [13], [14], that the primary electrophysiological structures present during sleep (including sleep spindles and hippocampal sharp-wave ripples) are temporally organized by the UDS [15], [16], [17], [18], and that UP-transitions generate precise spike patterns [19]. Thus, understanding UDS will likely yield insights not only into the cellular and network dynamics at play during slow-wave sleep, but also into the role of slow-wave sleep in memory formation.

UDS have most often been defined in terms of the MP of individual neurons, however given that robust spiking of both excitatory and inhibitory neurons occurs nearly exclusively during the UP state, classification of UDS based on extracellular unit activity is also possible [20], [21], [22], [23]. Due to their synchronous nature, large amplitude fluctuations in the LFP can also be used to classify UDS [12], [14], [24]. Classifying UDS from extracellular signals (multi-unit, LFP, EEG) offers numerous advantages, such as the data are easier to acquire, and such measurements can be done chronically, during natural behavior, relatively less invasively, and even in humans. Extracellular signals also present a natural means for estimating the ‘collective’ state of an ensemble of neurons. Moreover, due to the increasing popularity of multi-electrode recording methods, there is need for a general framework for classifying UDS based on extracellular signals.

Several different approaches to classifying UDS from LFP signals have been put forth uitilizing different features of the LFP signal. Mukovski et al. [25] argued that the high-frequency (20–80 Hz) power measured in a suitably localized window of time provides the best signal feature for classifying UDS, while more recently Saleem et al. [26] proposed using the low-frequency (<4 Hz) phase of the LFP for classification. While these works also differed in their method of inferring UDS from these signal features, both approaches were based on determining a fixed threshold (or set of thresholds) and comparing the signal feature(s) to these fixed threshold(s). We broadly term such approaches ‘threshold-crossing’ algorithms, and argue that the framework of hidden Markov models (HMMs) provides significant advantages over threshold-crossing methods for classifying UDS. In particular, HMMs provide a consistent, flexible, and statistically principled framework for inferring UDS from different types of signals that handles variations in experimental parameters such as type of anesthetic, type of electrode, precise electrode location and depth of anesthesia without the need for supervision, and can easily be adapted to accommodate non-stationarities in the data [27], [28]. The efficacy of HMMs for inferring UDS from stationary point-processes (extracellular spike times) has already been demonstrated [21], [22]. Here we describe an explicit-duration HMM (EDHMM) method for inferring UDS from potentially non-stationary sets of continuous signals (including MPs, LFPs, and EEGs), as well as a procedure for evaluating different signal features for UDS classification. This procedure shows that the low-frequency amplitude of the LFP provides the most information about the cortical state in our data. We demonstrate that the proposed method is very flexible and can also be applied effectively to classify UDS from EEG signals. We then show by comparing UDS inferred from simultaneously recorded LFP and MP signals that our EDHMM procedure produces significant improvements over standard methods.

## Methods

### Experimental Methods

#### Ethics statement

All surgical procedures and experiments were conducted according to the animal welfare guidelines of the Max Planck Society. The protocol was approved by the responsible State Committee on the ethics of animal experiments Karlsruhe (Permit Number 35-9185.81). All efforts were made to minimize suffering.

#### Animals, surgery, and histology

Methods were similar to those described previously [12], [24]. Briefly, MP and LFP data were obtained from 11 C57BL6 mice aged postnatal day p29 to p35, weighing between 16 and 23 g. Mice were anesthetized with urethane (1.7–2.0 g/kg i.p.). Body temperature was maintained at 37°C with the help of a heating blanket. The animal was head-fixed in a stereotaxic apparatus and the skull exposed. A metal plate was attached to the skull and a chamber was formed with dental acrylic which was filled with warm artificial cerebrospinal fluid. A 1 to 1.5 mm diameter hole was drilled over the left hemisphere and the underlying dura mater was removed. After electrophysiological recordings, mice were transcardially perfused with 0.1 M PBS followed by 4% paraformaldehyde, and 150 µm thick saggital brain sections were processed with the avidin–biotin–peroxidase method.

#### Electrophysiology and data acquisition

LFPs were recorded with a quartz/platinum-tungsten glass coated microelectrode (Thomas Recording GmbH, Giessen, Germany). *In vivo* intracellular membrane potential (MP) was recorded in whole-cell configuration by using borosilicate glass patch pipettes with DC resistances of 4–8 MΩ, filled with a solution containing 135 mM potassium gluconate, 4 mM KCl, 10 mM Hepes, 10 mM phosphocreatine, 4 mM MgATP, 0.3 mM Na3GTP (adjusted to pH 7.2 with KOH), and 0.2% biocytin for histological identification. Whole-cell recording configuration was achieved as described previously [29]. Relative to bregma, both the MP and the LFP recordings were made either around 1 to 1.5 mm anterior and 1 to 1.5 mm lateral (frontal) or around 3 mm anterior and 1 to 1.5 mm lateral (prefrontal). MP was recorded from pyramidal neurons at various depths, and LFP was recorded from upper layer 5. The recording site of the LFP was less than 1 mm distance from the neuronal soma from which the MP was obtained Both LFP and MP were recorded continuously on an eight-channel Cheetah acquisition system (Neuralynx, Tucson, AZ) for at least 600 s per experiment. That complete recording was used for subsequent analysis as described below. The MP was acquired by Axoclamp-2B (Axon Instruments, Union City, CA) and fed into a Lynx-8 amplifier (Neuralynx). LFP was sampled at 2 kHz, low-pass filtered below 475 Hz, and amplified 2,000 times. LFP signals were inverted so that UP states corresponded to positive deflections. MP was low-pass filtered below 9 kHz, sampled at 32 kHz, and amplified 80–100 times.

A total of 21 LFP recordings from 11 animals were analyzed. Usually a pair of LFP recordings were performed in a single animal, the recordings being separated by 50 to 210 minutes. Statistics were computed across LFP recordings. In addition, 9 MP recordings were performed simultaneously with 9 of the LFP recordings, all from different animals.

We recorded EEGs from 3 additional mice anesthetized with urethane (1.7–2.0 g/kg i.p.) and head-fixed in a stereotaxic apparatus. Two small incisions in the skin were made for subsequent insertion of recording electrodes. EEG signals were recorded with 150 µm diameter insulated silver wires, with exposed and chlorided tips inserted under the skin above the skull overlying left parietal cortex. The reference for this signal was taken from an identical wire inserted under the skin in the neck region above the left occipitum. This EEG was recorded with a Cheetah acquisition system as described above, sampled at 2 kHz, low-pass filtered below 400 Hz, and amplified 10,000 times.

### Statistical Methods

#### Hidden Markov model for UP and DOWN states

The problem of inferring the UP and DOWN state sequence given some measure of neural activity is well suited for the framework of HMMs [21], [22]. In a HMM, a sequence of data is modeled as being generated probabilistically from an underlying discrete-valued stochastic process [30], [31]. The observed data can be either discrete- or continuous-valued, while the unobservable ‘hidden’ state is a discrete random variable that can take *K* possible values (in our case two, representing the UP and DOWN states). Here we focus on inferring UDS from continuous signals such as the LFP or MP which are discretely sampled in time. Thus, we consider a time series of continuous-valued signal features 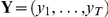 (e.g. the amplitude of a filtered LFP) extracted from the original signal, such that **Y** carries information about the underlying UP and DOWN states. Henceforth we will refer to **Y** as the ‘observation sequence’. Let 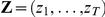 represent the sequence of hidden state variables, whose value at discrete time step *t* is given by *z_t_*. The HMM makes the simplifying assumption that **Z** is a first-order Markov chain [30], [31], which is characterized by *z_t_* being independent of the preceding sequence of hidden variables, given the value of *z_t−1_*. We can thus write
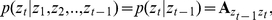
(1)where **A** is a matrix of state transition probabilities. The observation 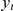 at time *t* is assumed to be conditionally independent of previous values of **Y** given *z_t_*, and is determined by a state-conditional observation distribution:
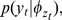
(2)where 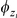 is a vector of model parameters for state 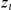.

In the general case we can define a vector-valued time series of observations 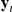. We use Gaussian observation distribution models of the form 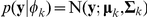, where 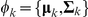 are the mean and covariance matrices associated with each hidden state *k*. Other observation distribution models could also be considered (in particular, mixtures of Gaussians have been used extensively for other applications) if the state-conditional observation distributions are determined to be highly non-Gaussian. Because some of the signal features characterizing the UP and DOWN states often change substantially over the course of the recording [27], [28], we allow the mean vectors to be (slowly varying) functions of time. While we do not consider such cases here, 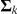 and 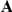 could also be allowed to vary slowly in time using a similar approach. Therefore, the Gaussian-observation HMM is fully specified by the parameter vector 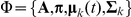, where 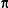 is a distribution over the initial hidden state variable *z_1_*. Signal features which are assumed constant in time are easily handled in this framework by simply restricting the 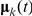 to be constants.

Maximum likelihood estimates of the model parameters 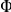 given a sequence of observations can be determined using the expectation maximization (EM) algorithm [31], [32] which proceeds using a two-step iteration. First, initial values for the parameters 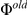 are selected, and the posterior distribution of the latent state sequence **Z** given the observation sequence **Y** and the parameter vector is computed. The posterior distribution of the latent state sequence is then used to compute the expected complete-data log likelihood [33]:
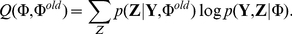
(3)Maximization of *Q* with respect to 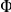 gives the new estimate of the parameter vector 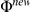. The process is repeated iteratively until convergence. For a given parameter vector, calculation of the posterior distributions of the latent variables is achieved using a set of recursions known as the forward-backward algorithm [31].

#### Explicit-duration HMM

An important weakness of the standard HMM is that it implicitly assumes a geometric distribution of state durations [31]. Numerous extensions of the standard HMM have been developed to address this issue, but the most frequently used is the explicit-duration HMM (EDHMM) [34], [35], which is a type of hidden semi-Markov model (for review see [36]). In the discrete-time EDHMM, the state duration is assumed to be a random variable which is restricted to take integer values in the range [1, *d_max_*], where *d_max_* is the maximum allowable state duration. Upon transitioning to a state *k*, a sequence of observations of length *d* will be emitted from the observation model *φ_k_*, with the observations assumed conditionally i.i.d. Each state can thus be specified by the pair (*k*,*d*), and state transitions are then determined by two such pairs 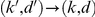. In the EDHMM, the simplifying assumption is made that 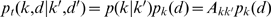 [36]. Thus, the probability of observing state *k* at time *t* depends only on the previous state *k′*, and the probability of state *k* having duration *d* depends only on the duration distribution *p_k_*(*d*) for state *k*. Since self-transitions are prohibited in the EDHMM, the transition matrix **A** is uniquely determined in the two state case:
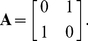
(4)


#### EDHMM parameter inference

Inference of model parameters in the EDHMM can be accomplished using a forward-backward algorithm similar to the standard HMM [34]. Yu and Kobayashi [37] demonstrated an efficient forward-backward algorithm for the EDHMM, and showed how to redefine the forward and backward variables in terms of posterior probabilities to avoid numerical underflow [38]. Defining 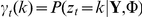 to be the marginal probability of state *k* at time *t* given the observation sequence **Y** and the model parameters 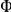, the observation model parameters are updated in the M step of the EM algorithm, in direct analogy with the estimation formulas in the standard HMM, according to:
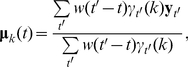
(5)

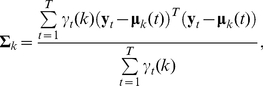
(6)where *w*(*t′−t*) is a symmetric, time-localized window function with a time-scale chosen to reflect the time-course of fluctuations in the state-conditional means.

#### State duration distributions

Defining 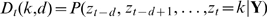 to be the conditional probability of state *k* starting at time *t-d* and ending at time *t* (lasting for duration *d*), Ferguson [34] showed that maximum likelihood estimates for the non-parametric distribution for state *k* are given by:
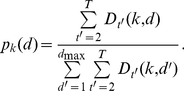
(7)Following Levinson's [35] use of a (continuous) parametric state duration model, Mitchell and Jamieson demonstrated how to find maximum likelihood solutions for the parameters of any exponential family distribution [39]. We follow this approach and use the two-parameter gamma and inverse Gaussian exponential family distributions to model the state durations. The (censored) discrete gamma distribution is given by:
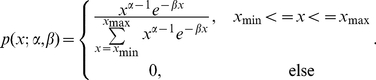
(8)The censored discrete inverse Gaussian distribution is given by:
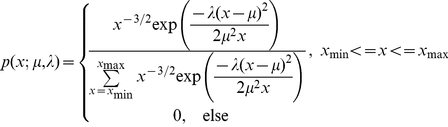
(9)Writing these two-parameter pmfs in the exponential family form gives:
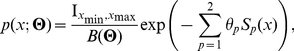
(10)where 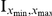 is the indicator function which is 1 inside the range [x_min_,x_max_] and 0 elsewhere, 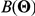 is a normalization constant, 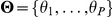 is the vector of P natural parameters, and 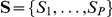 is the vector of natural statistics. Mitchell and Jamieson showed that maximum likelihood estimates for the natural parameters 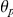 are given by solving the equations [39]:
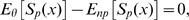
(11)where 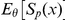 is the expectation of the *p* ^th^ natural statistic with respect to the exponential family distribution, and 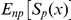 is its expectation with respect to the non-parametric distribution. We use Matlab's routine *fsolve* to solve for the maximum likelihood estimates of the parameters 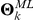 for each state *k*.

#### Maximum likelihood state sequence

Estimation of the maximum likelihood state sequence is typically achieved using the Viterbi algorithm [31], and similar algorithms have been demonstrated for the EDHMM [40]. We follow the method of Datta et al. [41], and map the Viterbi decoding problem into that of finding the longest path in a directed acyclic graph (DAG).

#### Discontinuous data segments

We can easily adapt the EDHMM to handle discontinuous segments of data, which can be useful if we wish to exclude certain portions of the data from analysis (for instance during periods of ‘desynchronized activity’ [42], [43], [44]). Assuming that we have *N_s_* discontinuous segments of data for which we wash to classify UDS, we define our segmented observation at time sample *t* within the *n* ^th^ segment to be 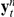. First, we must replace 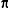 (the distribution on the initial latent state variable) with a matrix containing a distribution over the initial latent state variables for each data segment. Next we introduce a set of time-varying state-conditional mean functions, one for each data segment, which are updated according to:
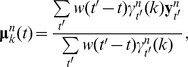
(12)The same forward-backward procedure can then be used to compute 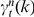 and 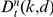 for each data segment independently. The time-invariant model parameters can be updated by computing expectations with sums over time samples and segments. For example, the updated estimate for the conditional covariance matrix of state *k* is given by:
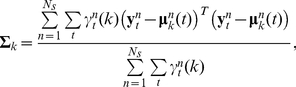
(13)where *N_s_* is the number of data segments. Computing the most likely state sequence within each data segment can be accomplished using the same Viterbi decoding algorithm to infer the maximum likelihood state sequence of each data segment independently. It's important to note that this approach implicitly assumes that the first and last state within each segment start and stop at the beginning and end of those segments respectively. These assumptions can be relaxed [36], however when the data segments are long relative to the state durations, the boundary states can be excluded from analysis without a significant loss of data.

#### Robustness to deviations from the observation model

Large deviations of the data from the observation model can lead to errors in inferring the state sequence, and thus the results were monitored for such deviations. While the model is not expected to be a perfect description of the data, large sudden changes in the signal properties, such as could be generated by movement artifacts [27], can create situations where the observation likelihood is very small under both state-conditional observation models. In such situations, where the observed data is very far from both state-conditional means, the posterior distribution on the hidden state will tend to be dominated by the state with highest variance. In order to avoid large negative deflections being attributed to the UP state or large positive deflections being attributed to the DOWN state, such instances were identified, and a more robust observation model was employed. Specifically, in such rare instances we use state-conditional Gaussian observation models where the state-conditional variances were constrained to be equal. This produces a more robust model which will always favor the hidden state whose mean is closer to the observed data at times when the data is very far from either state's mean.

#### Alignment of the decoded state transition times

In some cases, such as when the signal features are down-sampled or filtered, it is desirable to consider alignment of the initially decoded state transition times to a separate observation sequence, such as the less filtered or decimated data. This procedure allows for initial classification of the state sequence using an observation sequence with a lower sampling frequency *f_s_*, while subsequent alignment of the state transition times to a signal with higher *f_s_* prevents loss of temporal precision. This can be particularly important for the EDHMM where the complexity of the inference procedure and the Viterbi decoding algorithm both scale with *f_s_* ^2^ [37], [41] for a fixed maximum state duration. Alignment of state transition times to a new observation sequence can also be useful for removing the ‘blurring’ effects of filtering on the detected state transition times.

**Figure 1:**
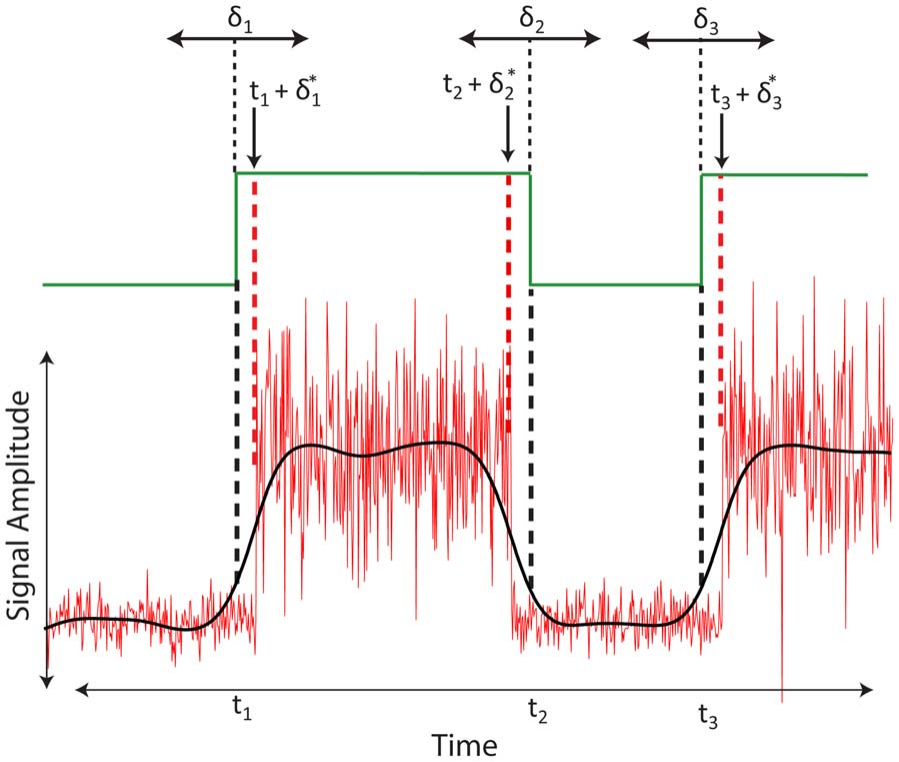
Schematic depiction of the maximum likelihood alignment procedure. An imaginary ‘two-state’ signal is shown in red. The low-pass filtered observation sequence (black trace) is used to produce the initially decoded Viterbi state sequence (green trace) with state transition times t_1_, t_2_, and t_3_ indicated by the vertical black dashed lines. These transition times are aligned to the broadband observation sequence (red trace) by maximizing the likelihood of a set of perturbations δ_i_ on the initial state transition times given the broadband observation sequence. Maximum likelihood values of the aligned transition times are then given by the set of times {t_i_+δ_i_*}, indicated by the red vertical dashed lines. For the signal features used in the majority of the analysis, perturbations of up to ±150 ms were determined to be sufficient; however when exploring a large range of filtering parameters we allowed perturbations of up to ±300 ms on the state transitions.

We perform this alignment using a dynamic programming algorithm to find the maximum likelihood set of state transition times given the new observation sequence and the model parameters. Let 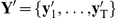 denote the ‘new’ observation sequence to which we want to align the state transition times. We first estimate the parameters of the state-conditional Gaussian observation distributions for **Y**′ using the posterior probabilities 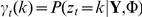 computed from the EDHMM fit to the initial observation sequence **Y**. Next, let 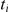 be the time of the transition into the *i* ^th^ state whose latent state variable is given by 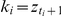, and let 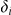 be a perturbation on the time of the transition into the *i* ^th^ state taking values in the range 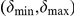 (figure 1). If we define 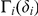 to be the maximized log likelihood of perturbations 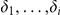 up to the *i^th^* transition (ignoring terms independent of the 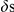 ) then we have the recursion relation:
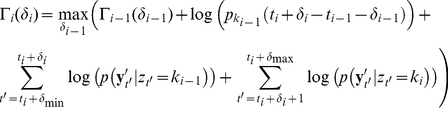
(14)By keeping track of the values of 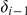 which maximize 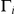 for each 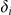 we can then backtrack the entire maximum likelihood sequence of transition perturbations, as with the Viterbi algorithm. We only need to include the initialization condition:
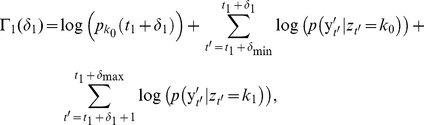
(15)where *k_0_* = *z_1_*. Seamari et al. achieved a similar realignment of state transition times by finding local maxima of the rate of change of the signal features in time [27]. Such a procedure will generally produce similar results to the maximum likelihood realignment procedure presented here, however additional ‘thresholding’ parameters may be required [27], and depending on the signal feature being used it may be more susceptible to noise.

#### EDHMM parameter initialization

It is well known that proper initialization of the model parameters is important to insure that the parameter estimates of the EM algorithm represent a global maximum of the likelihood function [31]. Several steps were thus taken to initialize the model parameters near their maximum likelihood values. First, the time-varying state means were initialized using a sliding-window density estimator. Specifically, Gaussian kernel density estimates were computed in overlapping, sliding windows of length *L*. The bandwidth of the density estimator was selected using Terrel's oversmoothing bandwidth selector [45]. If the density estimate was bimodal, the two modes were taken as the estimates of the UP and DOWN state means for the given interval. In cases where the density estimate was unimodal, the sign of the skewness of the density was used to determine whether the single mode was representing the UP state or the DOWN state (positive skewness indicating the mode representing the DOWN state and vice versa). In such cases, the mean of the other state was taken to be:
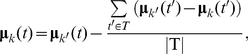
(16)where 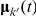 is the mean estimate for the state *k′* representing the mode of the density estimate at time *t*, and the set 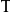 includes all times for which the density estimate was bimodal. Recordings in which the density estimate was never, or very rarely, bimodal (and which still met power spectral criteria for the presence of UP-DOWN states) were not considered here, however other initialization procedures could be introduced in such cases. The state covariance matrices 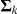 were then initialized by fitting Gaussian mixture models to the variables 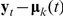 for each state *k*. Similar results were obtained by fitting sliding-window Gaussian mixture models to the observation sequence, however this was more computationally expensive.

After initializing the observation model parameters as described above, the initial transition matrix **A** was set to:
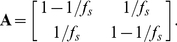
(17)This initializes the expected state durations for both the UP and DOWN states to be 1 s under a geometric state duration distribution. A standard HMM was then fit to the observation sequence using this set of initial parameters. Next, the maximum likelihood state sequence was estimated using the standard Viterbi decoding algorithm, and the set of state durations 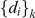 for each state was computed from the Viterbi state sequence.

Initial values for the parameters of the state duration distributions were then computed from the Viterbi state durations 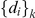 of the HMM. For the gamma distribution, maximum likelihood estimates of the state-dependent shape parameters α_k_ were determined numerically using the Newton-Raphson method [46]. The rate parameters *β_k_* are then given analytically by:
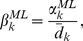
(18)where 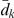 is of the sample mean duration of state *k*. For the inverse Gaussian distribution, ML estimates of the model parameters are given by:
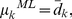
(19)

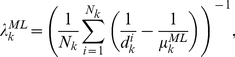
(20)where *N_k_* is the number of occurrences of state *k*. The ML estimates of the observation distribution parameters and the state duration distribution parameters determined from the HMM were used to initialize the parameters of the EDHMM.

#### EDHMM method summary

**Figure 2:**
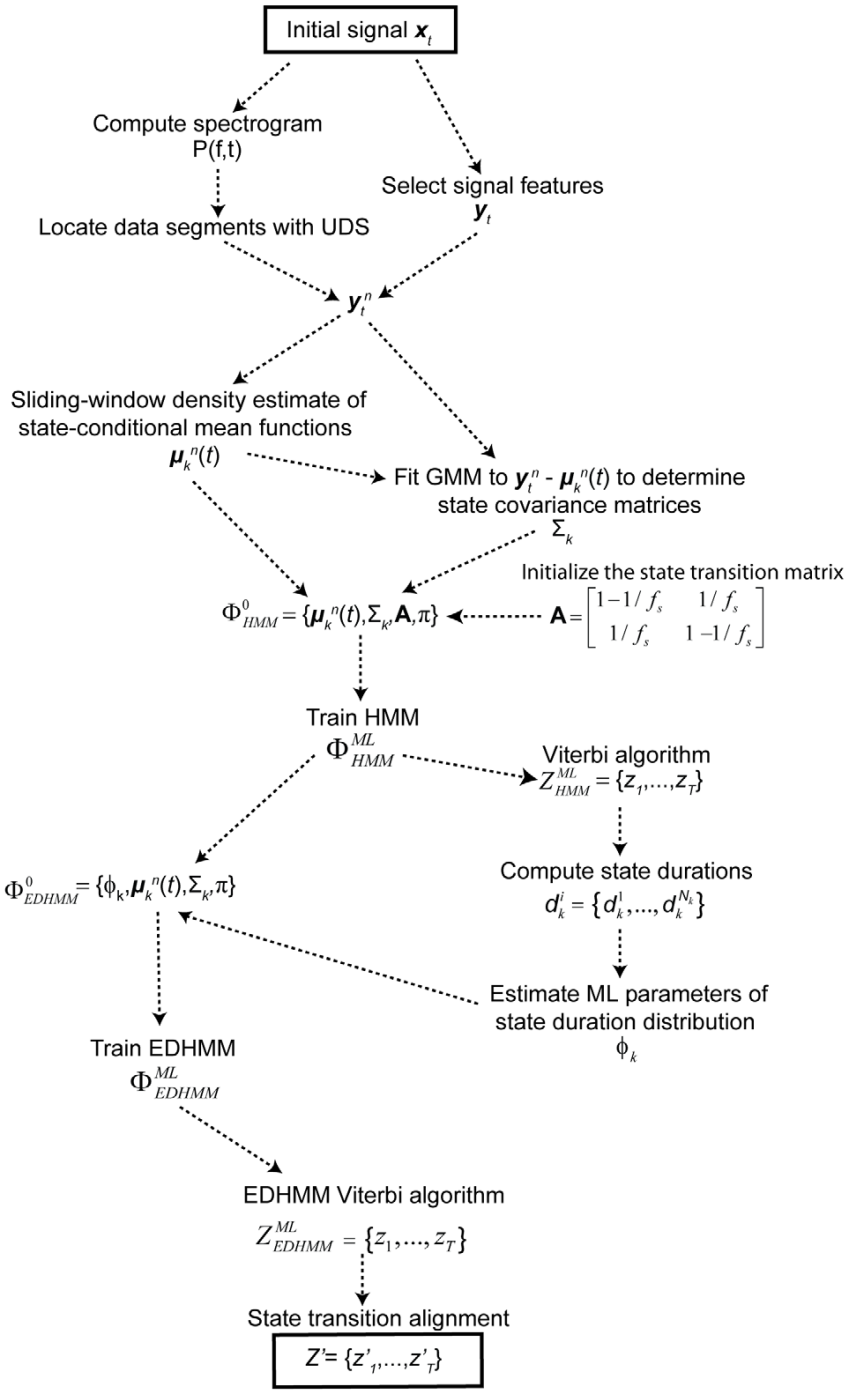
Flow diagram of the EDHMM inference algorithm. The full procedure for UDS inference from continuous signal features is depicted schematically. The initial input to the algorithm is the raw signal ***x*** *_t_*, while the final output of the algorithm is the Viterbi state sequence of the EDHMM ***Z′***, aligned to the broadband signal.

In summary, the entire EDHMM UDS inference algorithm proceeds as follows (figure 2). First, the segments of data meeting the criteria for the presence of UDS were extracted. Then, signal features (the observation sequence) were calculated and down-sampled to a sampling frequency of 50 Hz within each segment. Initial estimates of the time-varying state means were computed using the sliding window kernel density estimator. Gaussian mixture models fit to the difference of the observed data and the time-varying mean estimates were then used to initialize the state-conditional covariance matrices. Next, maximum likelihood estimates of the parameters of a standard HMM were determined, and were used to compute the Viterbi state sequence of the HMM. The state durations of the Viterbi state sequence were used to estimate the maximum likelihood parameters of the state duration distribution models. These, along with the ML estimates of the observation model parameters and the matrix of initial state probability distributions for all data segments from the HMM, were used to initialize the parameter vector for the EDHMM. Using a maximum state duration of 30 s, only a few iterations of the EM algorithm were typically sufficient to achieve convergence of the log likelihood with a tolerance of 1e-5 for the EDHMM. The most likely state sequence under the EDHMM was then determined, and subsequent alignment of the state transition times to the broadband signal sampled at 252 Hz was performed by maximizing the likelihood of a sequence of perturbations on the transition times. Code was written in Matlab (The MathWorks, Natick, MA). Algorithms for training HMMs were modified from the HMMBOX Matlab toolbox (available at http://www.robots.ox.ac.uk/~irezek/software.html). Algorithms for training EDHMMs were modified from source code provided by Shun-Zheng Yu [38] (available at http://sist.sysu.edu.cn/~syu/SourceCode.html). The full algorithm was found to take on average 7 s per minute of raw data for UDS inference from scalar signal features (run using Windows 7 64-bit with an Intel Core i7 2.67 GHz processor and 6GB of RAM). When using the simpler HMM, without explicit state-duration modeling, the algorithm took about 1 s per minute of raw data on average. A Matlab implementation of the algorithms described is available upon request.

#### Signal separability

The Bhattacharyya distance measures the similarity of two probability distributions *p* and *q*, and is given by [47]:
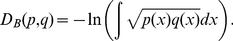
(21)
*D_B_* is frequently used as a criterion for feature selection in the context of classification problems [48], as it measures the separability of the component distributions. In the case where the distributions *p* and *q* are multivariate normal distributions, the Bhattacharyya distance is given by [48]:
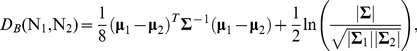
(22)where 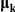 and 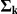 are the mean and covariance matrix of the *k* ^th^ Gaussian component, and 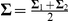. For comparing Gaussian distributions with time-varying means we use the time-averaged Mahalonobis distance, giving:
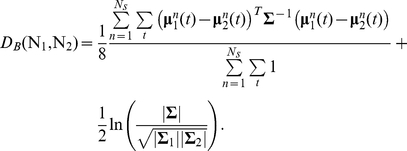
(23)


#### MP-LFP UDS correspondence

In order to compare UDS classified from simultaneously recorded LFP and MP signals in the same brain region (e.g. the frontal cortex), we assigned a best correspondence between individual LFP and MP UP and DOWN states. This allowed us to determine the probability of detecting spurious UP or DOWN states in the LFP not present in the MP, as well as the probability of missing UP or DOWN states in the LFP that were present in the MP. To assign such a correspondence, LFP and MP UP transitions were first linked using a greedy search algorithm. In each iteration of the algorithm the LFP-MP UP transition pair that was least separated in time was linked. This procedure was terminated once either all of the LFP or all of the MP UP transitions had been linked. Next, any crossed links were eliminated by removing the link between the UP transition pair that was more separated in time. This served to preserve the same temporal ordering of the LFP UP transitions and their linked MP counterparts. After assigning corresponding UP transitions, a similar greedy search algorithm was used to link LFP and MP DOWN transitions. As with UP transition assignments, crossed linkage of DOWN transition pairs was not allowed. After this procedure, pairs of LFP state transitions which were not matched to any MP state transitions were labeled as ‘extra’ LFP states, while MP state transitions which were not matched to any LFP state transitions were taken as ‘missed’ states.

## Results

### Non-stationarities in the UDS

**Figure 3:**
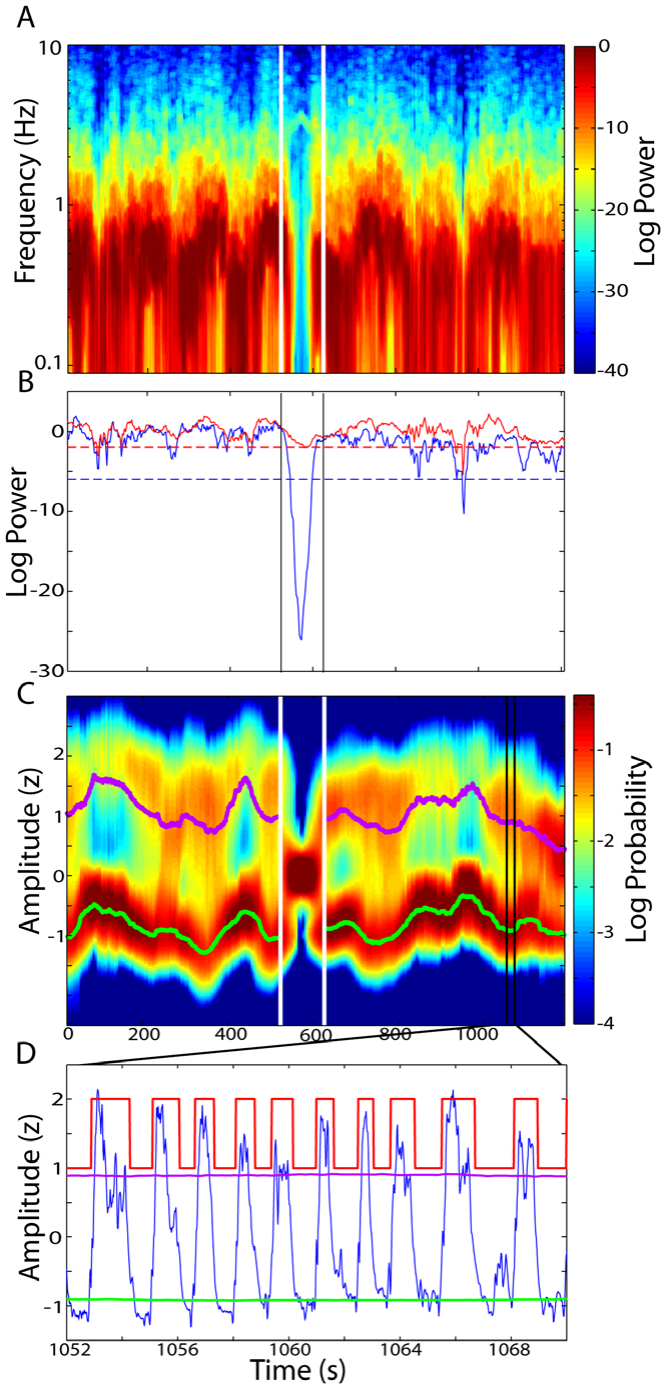
Procedure for selecting data epochs with clear UDS. **A**) The multi-taper spectrogram of a cortical LFP. White vertical lines indicate a period of desynchronized activity with decreased power in the low-frequency (UDS) range. **B**) The maximum power in the UDS range (0.05–2 Hz; blue trace) and the high-frequency power (4–40 Hz; red trace) are extracted from the spectrogram. Threshold values (dashed lines) for each of these statistics are used to locate data segments that have insufficient UDS power while having substantial high-frequency power. These desynchronized epochs (highlighted by the vertical lines) are excluded from all analysis. **C**) The sliding-window density estimate of the low-frequency (0.05–2 Hz) LFP amplitude from the same recording is plotted along with the time-varying UP (violet) and DOWN (green) state-conditional means estimated from the HMM. The period of desynchronized activity (vertical white lines) shows a loss of bimodality. **D**) An example LFP trace (blue) taken from the region of the recording indicated by the black lines. The violet and green traces again represent the time-varying UP and DOWN state-conditional means. The red trace shows the inferred UDS state sequence.

It has been well documented that the UDS seen under anesthesia are sometimes interrupted by so-called desynchronized epochs [42], [43], [44], which are periods lacking clear UDS. Thus, for all our analysis we first located the epochs of data containing clear UDS based on the spectral properties of the signal (figures 3A,B). The spectrogram of the z-scored signal was computed in 15 s overlapping windows using multi-taper methods (Chronux Matlab toolbox [49]; http://chronux.org) with a time-bandwidth product of 4, and 7 tapers [50]. The maximum power in the range 0.05–2 Hz (‘UDS power’) was then computed for each signal, along with the integral of the log power between 4 and 40 Hz (‘reference power’). These statistics were used to test for the presence of clear UDS in each segment. A single threshold value was set for the ‘UDS’ and ‘reference’ power based on visual inspection across recording sessions (figures 3A,B). Any data segments which had UDS power below this threshold and reference power above the threshold were excluded from analysis as desynchronized epochs. The reference power criterion insured that any segments of data which had low power in the UDS band because of very long DOWN states were not excluded from UDS analysis, since these segments would show a corresponding reduction in high-frequency power. All subsequent analysis was performed only for those segments of data containing clear UDS (see Methods). In all cases we inverted the LFP signal so that the UP states corresponded to positive deflections in order to facilitate comparison with membrane potential UDS.

Initially, we applied a Gaussian-observation HMM with two hidden states to classify UDS using the amplitude of the low-frequency (0.05–2 Hz) LFP [12], [24]. Thus, the distribution of this signal feature (‘observation’) within each hidden state (the UP and DOWN states) was modeled as Gaussian (see Methods). When classifying UDS over long duration recordings (∼20 minutes) we found that the amplitudes of the UP and DOWN states could vary substantially over the course of the recording [27], [28] (figure 3C). This could arise from actual changes in the amplitudes of the UDS, as well as filtering artifacts of the AC-coupled amplifiers. To account for variations in the UP and DOWN state amplitudes, we introduced time-varying state-conditional means into the model by using a sliding-window estimate of the state-conditional mean amplitudes (see Methods). Since each UDS cycle was about 2 s long, a window length of 50 s was found to provide a good tradeoff between temporal resolution and robustness. Thus, the state-conditional mean amplitudes at a given time were computed using the 50 s of data surrounding the time point.

### Choosing a state duration distribution model

**Figure 4:**
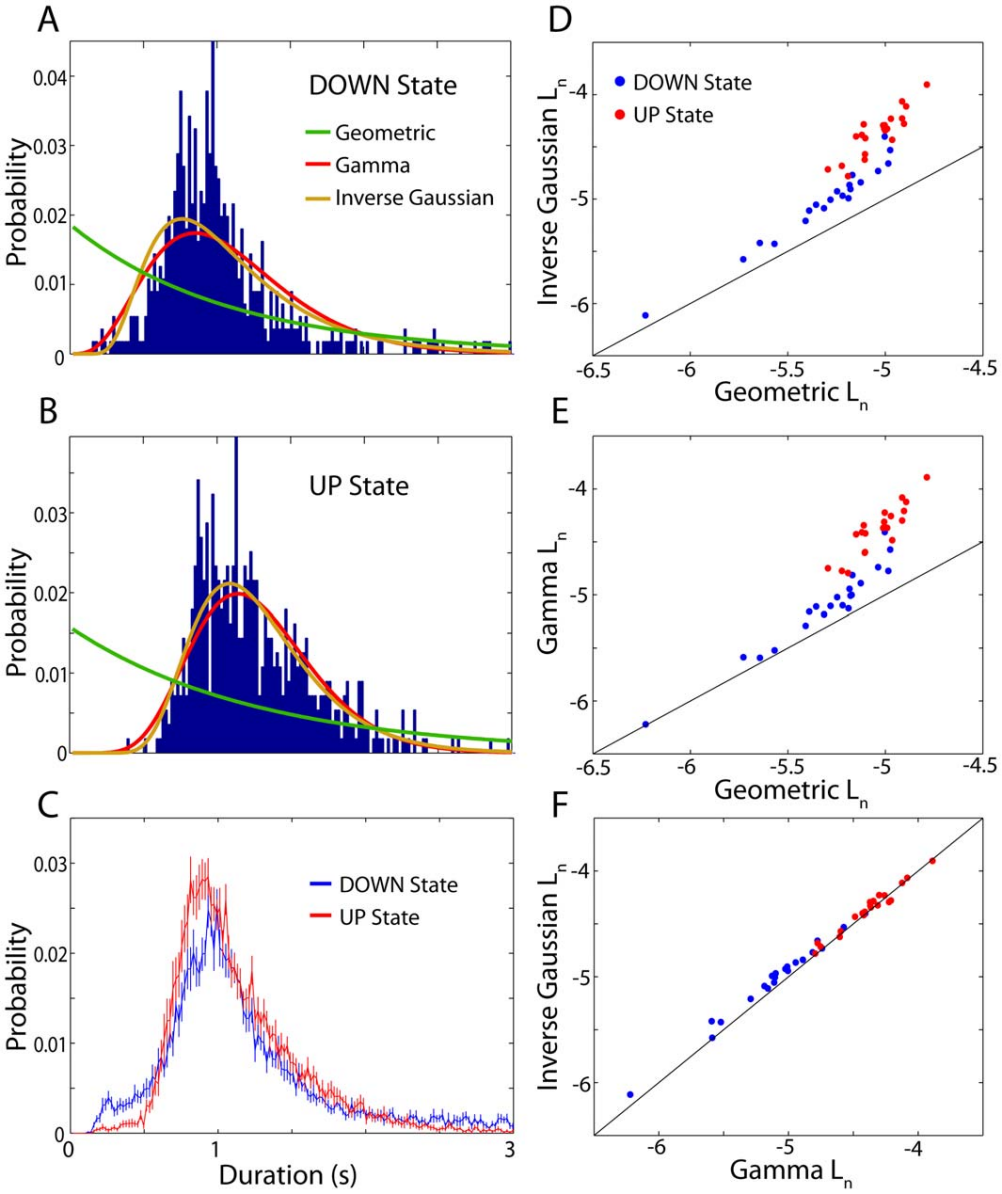
Explicit models for UP and DOWN state duration distributions. **A**) The distribution of DOWN state durations inferred by the HMM algorithm for an example LFP recording. The green trace shows the maximum-likelihood geometric distribution fit. The red and orange traces are the maximum likelihood fits for the gamma and inverse Gaussian distributions respectively, showing much better fits to the empirical distribution than the geometric distribution. **B**) Same as A for the UP state durations. **C**) DOWN (blue) and UP (red) state duration distributions averaged across all (n = 21) LFP recordings. Error bars indicate mean ± standard error of the mean. **D**) The maximized log-likelihood per sample *L_n_* for the geometric distribution is plotted against *L_n_* for the inverse Gaussian distribution across all LFP recordings for the DOWN (blue dots) and UP (red dots) state duration distributions. The inverse Gaussian distribution was a much better model for the data than the geometric distribution for both the DOWN (inverse Gaussian: median: −4.97, inter-quartile range: −5.14–4.82; geometric: −5.22, −5.39–5.16; *p* = 6.0e-5, two-sided Wilcoxon signed rank test), and UP (inverse Gaussian: −4.33, −4.47–4.27; geometric: −5.01, −5.11–4.95; *p* = 6.0e-5) states. **E**) Same as D for the gamma distribution. The gamma distribution also produced higher normalized log-likelihoods than the geometric distribution for both DOWN (−5.10, −5.21–4.87; *p* = 6.0e-5) and UP (−4.51, −4.37–4.25; *p* = 6.0e-5) state duration distributions. **F**) The inverse Gaussian distribution produced significantly better fits than the gamma distribution for the DOWN (p = 6.0e-5), as well as the UP (p = 0.027) states.

The distributions of UP and DOWN state durations obtained from the HMM were very far from the geometric distribution implicitly assumed by the HMM (figure 4). Thus, in order to determine an appropriate choice of duration distribution model, we computed the maximized log-likelihood per sample *L_n_* for several exponential family distributions including the gamma distribution and inverse Gaussian distribution, as well as the geometric distribution [21]. Both the inverse Gaussian (DOWN: *p* = 6.0e-5; UP: *p* = 6.0e-5; two-sided Wilcoxon signed rank test, n = 21 LFPs; figure 4D) and gamma (DOWN: *p* = 6.0e-5; UP: *p* = 6.0e-5; figure 4E) distributions produced far better fits than the geometric distribution for both the DOWN and UP state duration distributions. Furthermore, the inverse Gaussian distribution produced significantly better fits than the gamma distribution for both the DOWN (*p* = 6.0e-5) and the UP (*p* = 0.027) state durations (figure 4F). Similar results were obtained when using model selection criteria with a penalty for model complexity such as the Akieke information criterion (AIC) or the Bayesian information criterion (BIC). Thus, to improve upon the implicit use of the geometric state duration model, we use an EDHMM with an inverse Gaussian distribution model for both the UP and DOWN state durations.

### LFP feature selection

Thus far we have only considered inferring UDS from the amplitude of the low-frequency component of the LFP, but previous work [25] has shown that the signal power in the 20–80 Hz range can be more effective for inferring LFP UDS. Thus, we sought to compare results obtained using the low-frequency amplitude (LF-amplitude) and high-frequency power (HF-power). Furthermore, classification results depend on the preprocessing steps used to compute the LF-amplitude and HF-power of the LFP. Thus, if we believe (whether because of evidence accumulated from intracellular recordings, or because we have some knowledge about the underlying mechanisms governing state transitions) that rapid (e.g. <200 ms) fluctuations in the signal features are unlikely to represent transitions between states, we can apply this prior information by appropriately bandlimiting the signal features.

To obtain the ‘instantaneous’ high-frequency power we convolved the squared amplitude of the filtered signal with a Gaussian smoothing kernel. This smoothed power was then log-transformed in order to normalize the state-conditional distributions. We varied the high-cutoff frequency (HCF) and Gaussian smoothing sigma of the LF-amplitude and HF-power respectively to control the bandwidth of the signal features. Since such low-pass filtering of the signal features will also have a ‘blurring’ effect on the precise state transition times, we aligned the initially detected state transition times to a broadband signal by optimizing a sequence of perturbations on the state transition times (figure 1), analogous to the procedure used by Seamari et al [27]. This also allowed for more direct comparisons of the distributions of detected state durations when using various filtering/smoothing parameters.

**Figure 5:**
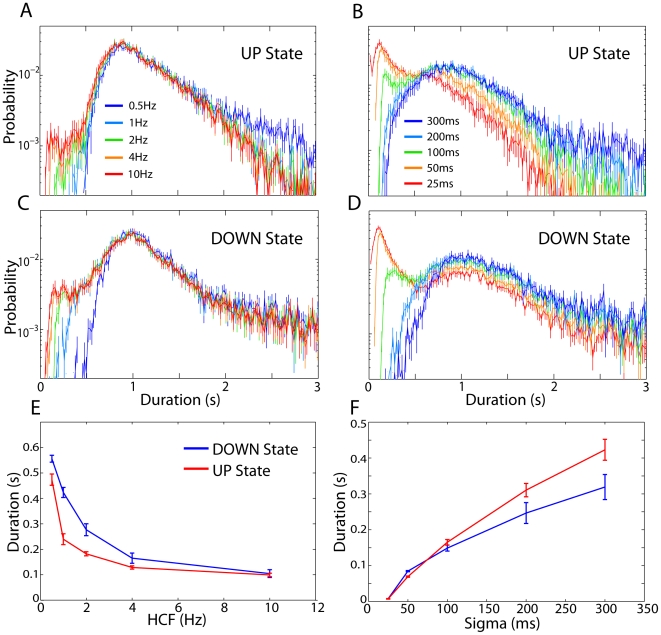
Dependence of UDS classification on signal preprocessing. **A**) The average UP state duration distribution for HMM classification based on the filtered LFP amplitude is plotted for several different values of the high-cutoff frequency (HCF). As the HCF increases from 0.5 Hz to 10 Hz, the UP state duration distribution starts to develop a secondary peak at short durations (<200 ms). **B**) Same as A for inference based on high-frequency LFP power using several different values of the Gaussian smoothing sigma. In this case the short duration peak is more pronounced than for classification of the low-frequency LFP amplitude, and it appears for smoothing windows as large as sigma = 100 ms. **C–D**) Same as A–B for the DOWN state duration distributions, showing similar effects of filtering and smoothing on the detected state durations. **E**) The minimum DOWN (blue) and UP (red) state durations, averaged across recordings (n = 21), is plotted as a function of the HCF. For HCFs as low as about 2 Hz, state durations as short as 200 ms are still detected. **F**) Same as E for the high-frequency power based classification. As the smoothing sigma is increased above about sigma = 150 ms the minimum state duration exceeds 200 ms.

First, we compare the state duration distributions obtained from an HMM fit to these different signal features. Figures 5A,C show that as the HCF of the LF-amplitude was increased from 0.5 to 10 Hz, the state duration distributions started to develop a secondary peak at short (<200 ms) durations. These short-duration states represent a deviation from the unimodal state duration distributions that cannot be well modeled by simple two-parameter distributions, and likely correspond to the spurious detection of state transitions. Furthermore, classification based on the HF-power produced far more short-lived states than when using the LF-amplitude, even when the smoothing sigma was as large as 100 ms (figures 5B,D). These results suggest that the frequency content of the signal features used for classification should be restricted to prevent excessive detection of short-lived states; however, when the signal features were excessively bandlimited the range of detectable state durations became overly restricted (figures 5E,F).

We thus chose HCF = 2 Hz and sigma = 150 ms to provide a good compromise between minimizing the presence of spuriously detected states while maximizing the range of ‘allowed’ state durations. Other authors [20], [21], [23], [25], [26], [27] have used threshold minimum state durations, typically in the range 100–200 ms, to avoid detecting such spurious state transitions; however such thresholds can produce ambiguous state sequences (if multiple states with subthreshold duration occur in sequence). When applied in the EDHMM framework, such thresholding can produce a large number of states whose duration is exactly equal to the minimum allowed duration. Our preprocessing of the signal features imparts a ‘prior’ bias against overly short state durations without imposing a hard threshold, thus avoiding these problems. It is worth noting at this point that the assumption of conditional independence of the observation sequence used in the HMM (and EDHMM) is clearly not strictly valid, particularly for the bandlimited signal features. The auto-regressive HMM (ARHMM) relaxes this assumption by modeling the signal features with state-dependent autoregressive models [51]. We found that despite this assumption, the HMM produced better results than an ARHMM for UDS classification (results not shown), likely because the UP and DOWN states are better distinguished by their state-conditional distributions than by their state-conditional autocorrelation functions.

Next, we computed the separability of the LFP LF-amplitude and HF-power, where separability was measured by the Bhattacharyya distance between the state-conditional Gaussian observation distributions computed for the EDHMM (see Methods). This measure is frequently used for feature selection and quantifies how separable the component distributions are for a given signal feature. We used the time-varying state-conditional means when estimating the separability so that variations in the state means did not decrease the apparent separability.

We found that the LF-amplitude provided significantly more separability than the HF-power (LF: median: 1.95, inter-quartile range: 1.83–2.07; HF: 1.30, 1.10–1.58; *p* = 6.0e-5), suggesting that for our data the LFP LF-amplitude was a more effective signal feature than the HF-power for inferring UDS. Furthermore, the HF-power provided significantly more variable separability across LFP recordings than the LF-amplitude (variances: LF: 0.016, HF: 0.11; *p* = 5.1e-5, two-tailed F-test for equal variances). We also computed the separability of the combined LF-amplitude and HF-power signals (LF+HF: 1.79, 1.72–1.90), and found that it was significantly less than in the LF-amplitude alone (*p* = 6.4e-3). Thus, using both LF-amplitude and HF-power simultaneously did not increase the separability of the state-conditional distributions.

Certainly, more ‘elaborate’ signal features, such as time-frequency representations (e.g. coefficients of the continuous wavelet transform) could also be used, but such approaches are not expected to provide substantial advantages since differences in the state-conditional power spectra are largely redundant across frequencies [25]. However, the EDHMM framework can be applied to any signal features which can be well modeled with state-conditional Gaussian (or mixture of Gaussians) distributions. Thus, for a given data set, various signal features should be evaluated to determine whether there is an appropriate two-state mixture distribution, and whether there is sufficient separability between the state-conditional distributions to perform robust inference of UDS.

### Comparison to threshold-crossing approaches

The results of the EDHMM UDS classification method are compared with those of a ‘fixed threshold-crossing’ (TC) approach, using the LF-amplitude (0.05–2 Hz). The fixed threshold was selected for each LFP recording using either a ‘static mixture model’ (SMM) method, or a ‘nonparametric’ (Np) method. For the SMM, we selected a threshold by fitting a two-component Gaussian mixture model to the signal feature. The threshold was then chosen to be the value of the signal in the range 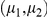 where the two mixture components had equal probability. For the ‘nonparametric’ approach, the threshold was chosen as the location of the local minimum of a Gaussian kernel density estimate of the signal in the range 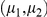 with the lowest value, similar to the method used by Mukovski et al [25]. In this case, the state means were again taken from the two-component mixture model fit. Given the threshold value, the threshold-crossing times were then used to identify UP and DOWN states.

**Figure 6:**
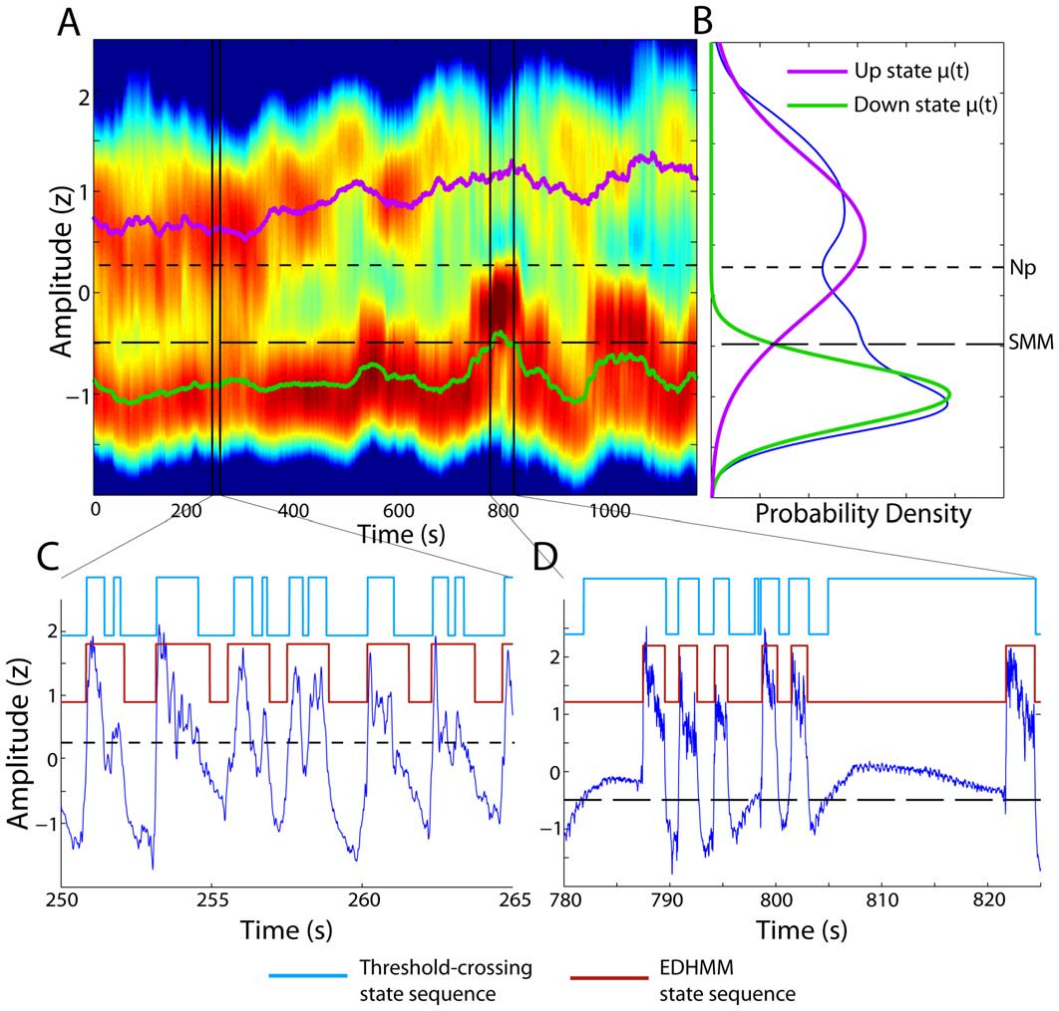
Comparison of EDHMM classification and threshold-crossing classification. **A**) The sliding-window density estimate of the LF-amplitude is shown for an example LFP recording. Log probability density is depicted as the color map, with the violet and green traces showing the time-varying state-conditional means. The regions indicated by the vertical black lines show the locations within the data where the example traces in panels C and D were taken. **B**) The overall amplitude distribution, along with the UP and DOWN state component distributions fit using a (static) Gaussian mixture model. The two values of the fixed threshold used for the “threshold-crossing” algorithm, chosen using the non-parametric (Np) and static mixture model (SMM) approaches, are shown by the finely and coarsely dashed black horizontal lines respectively. **C**) An example LFP trace (blue) from the region indicated by the black lines. The Viterbi state sequence from the EDHMM is shown in brown. For comparison, the state sequence classified using the nonparametric (Np) threshold-crossing method is shown in light blue. In this example, the threshold value appears to be too high, and many of the UP states are erroneously split by falsely detected DOWN states. **D**) Another example LFP trace from later in the recording. In this case, the state sequence classified using the SMM threshold-crossing method is shown in light blue for comparison. The SMM threshold appears to be too low in this example, producing a number of falsely detected UP states while the LFP clearly remains in the DOWN state.

While for the most part UDS can be inferred accurately using the TC methods, non-stationarities in the data, and ambiguous, intermediate amplitude signal features can pose substantial problems. Figure 6 illustrates some potential problems with the TC approach for an example LFP signal. In figure 6C, the threshold value selected by the Np method appears to be too high such that fluctuations within the UP state repeatedly trigger DOWN state transitions. The threshold value selected using the SMM method was substantially lower and could largely avoid these errors; however, the SMM threshold was found to be too low at other times within the same recording. As shown in figure 6D, at around 800 s there was a period with increased probability of the DOWN state which was also accompanied by an increased mean amplitude of the DOWN state, resulting from high-pass filtering of the long DOWN states by the AC-coupled amplifiers. The threshold value selected by the SMM method was found to be substantially too low during this period (figure 6D), such that a series of erroneous UP states were detected while the signal was apparently remaining in the DOWN state. Thus, the problem with the TC methods is often not one of selecting the ‘appropriate’ fixed threshold value, but rather that no single threshold value exists which can provide robust separation of the UP and DOWN states across the entire recording. The lack of an explicit or implicit state duration model also results in the increased detection of spurious short-lived UP and DOWN states when using TC methods. States lasting for less than 200 ms were much more prevalent when using TC methods (SMM: 1.9%, 1.6–2.8%; *p* = 6.0e-5; Np: 2.0%, 1.3–2.8%; *p* = 6.9e-5) compared to the EDHMM method (0.7%, 0.2–1.0%). Such short lived states were also more prevalent when using the simpler HMM method (0.9%, 0.4–1.4%; *p* = 5.4e-4), illustrating the importance of incorporating explicit state duration models.

**Figure 7:**
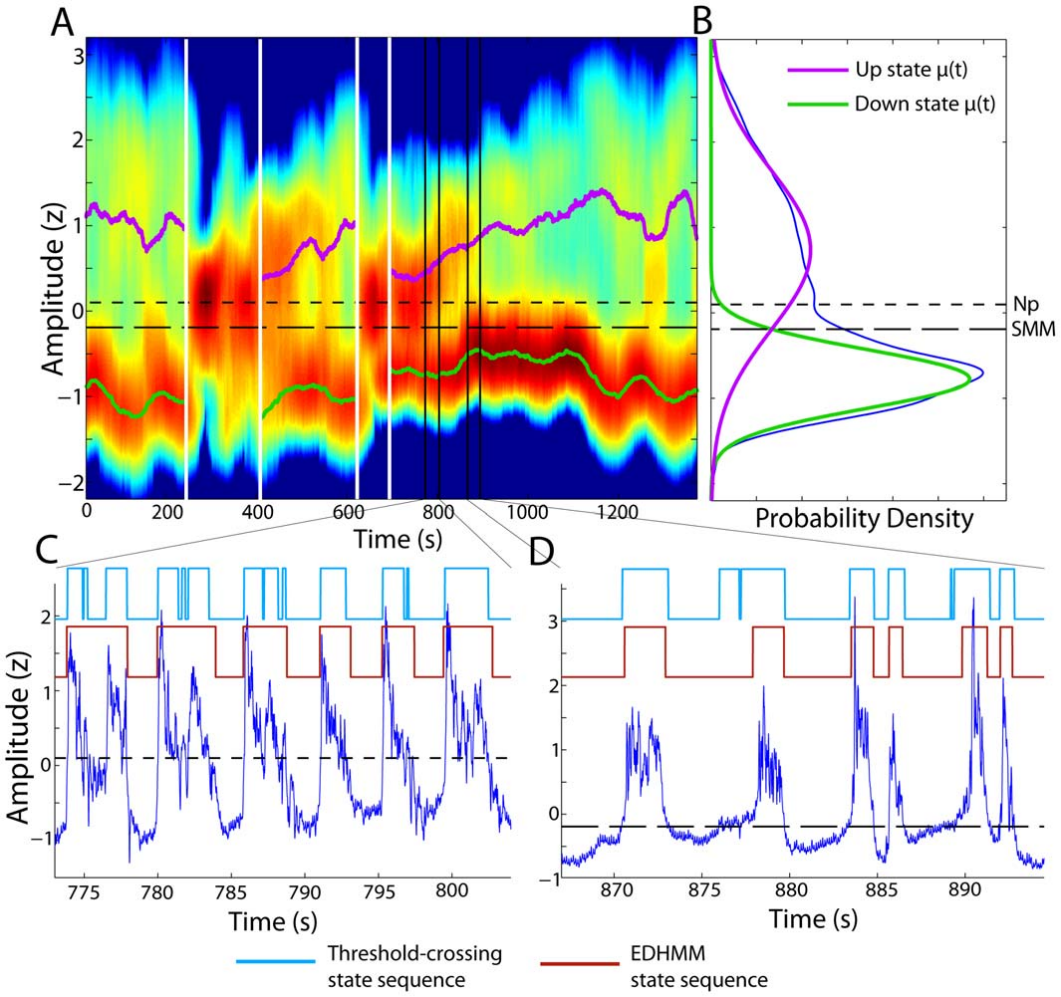
EDHMM UDS classification from a scalp EEG recording. **A**) The sliding-window density estimate of the LF-amplitude is shown for an example EEG recording. Log probability density is depicted as the color map, with the violet and green traces showing the time-varying state-conditional means. The regions indicated by the vertical black lines show the locations within the data where the example traces in panels C and D were taken, and the white vertical lines indicate desynchronized epochs. **B**) The overall amplitude distribution, along with the UP and DOWN state component distributions, fit using a (static) Gaussian mixture model. The two values of the fixed threshold used for the “threshold-crossing” algorithm, chosen using the Np and SMM approaches, are shown by the finely and coarsely dashed black horizontal lines respectively. **C**) An example EEG trace (blue) from the region indicated by the black lines. The Viterbi state sequence from the EDHMM is shown in brown. For comparison, the state sequence classified using the Np threshold-crossing method is shown in light blue. **D**) Another example EEG trace comparing the EDHMM UDS classification with the SMM threshold-crossing method.

In order to demonstrate the flexibility of the proposed algorithm, we also applied the EDHMM method to classify UDS from scalp EEG recordings. As with the LFP recordings, we found that the EDHMM method produced reliable classification of EEG UDS, even when the state-conditional distributions were non-stationary and/or not well separated. Figure 7 shows a comparison of UDS classified using the EDHMM method with classification using TC methods for an example EEG recording. Even though the EEG UDS properties changed substantially over the course of the recording (figure 7A), and the overall LF-amplitude distribution was not clearly bimodal (figure 7B), the EDHMM method produces a robust and consistent classification of UDS which was at times much better than the TC method using either threshold selection method (figures 7C–D). Hence, while a TC approach gave results which were mostly in agreement with those of the EDHMM method, the advantages of using the EDHMM inference procedure were particularly important when substantial non-stationarities were present, or when the UP and DOWN state-conditional distributions were not well separated.

### Evaluating classification accuracy

**Figure 8:**
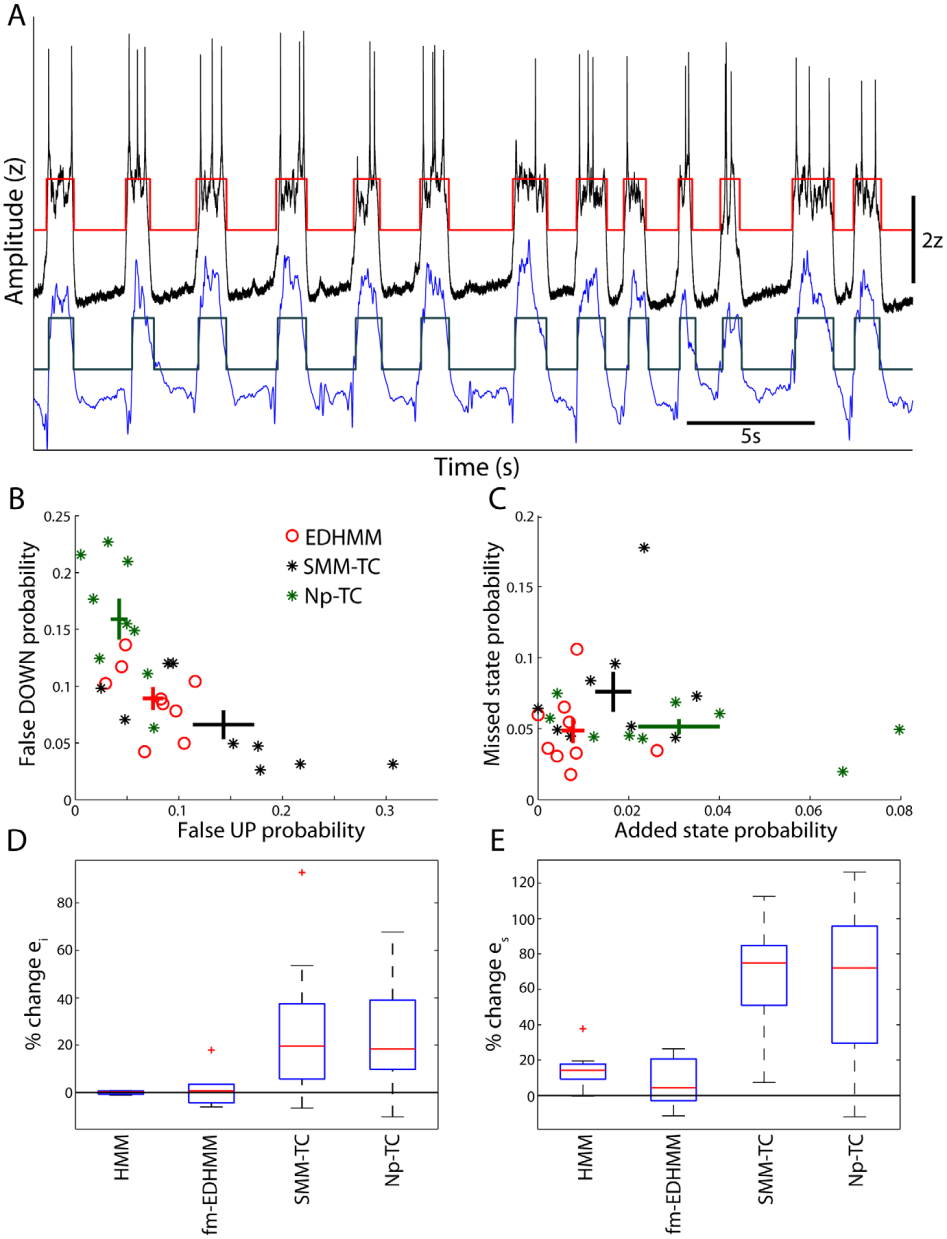
EDHMM classification produces improved agreement between simultaneously recorded LFP and MP signals. **A**) Example of an LFP (blue trace) recorded simultaneously with the MP (black trace) of a nearby cortical neuron. The corresponding LFP (gray) and MP (red) state sequences inferred from the EDHMM are overlaid. **B**) The instantaneous probability of detecting false DOWN states is plotted against that for false UP states for the EDHMM method as well as the SMM- and Np-TC methods. The mean and SEM are indicated by the colored crosses. **C**) The probability of a missed LFP state, relative to the MP state sequence, is plotted against the probability of detecting an extra LFP state. **D**) Box plots illustrating the changes in *e_i_* relative to the EDHMM algorithm for the following decoding algorithms: HMM; fixed-mean EDHMM (fm-EDHMM); static mixture model threshold-crossing (SMM-TC); and nonparametric threshold-crossing (Np-TC). **E**) Same as D for *e_s_*.

In order to quantitatively compare the results produced using the various methods and signal features described above, we compared the similarity between state sequences classified from the LFP and those classified from the simultaneously recorded MP of nearby cortical neurons, using two different similarity metrics (figure 8). The state of single cortical neurons is classified relatively unambiguously from the signal amplitude [25], [26], and is a reliable indicator of the cortical state since cortical neurons make nearly synchronous state transitions [52]. MP state sequences were computed from the LF-amplitude (0.05–2 Hz) after removing spikes from the MP signal. When considering different algorithms (e.g. threshold-crossing vs. EDHMM), identical methods were used to infer UDS in the MP and LFP to insure fair comparisons between methods. We first computed the instantaneous probability of error in the LFP state sequence relative to the MP state sequence, determining both the probability of detecting false UP and false DOWN states in the LFP. We take the sum of the false UP and false DOWN probabilities as a measure of the instantaneous error probability *e_I_*. As *e_i_* is sensitive to the precise relative timing of UDS, but is not necessarily sensitive to additions or deletions of states, we also compute a measure of the ‘state error’ probability *e_s_*. *e_s_* is defined as the sum of the probabilities of state additions and state deletions in the LFP UDS relative to the MP UDS, and is computed after determining the best correspondence between MP and LFP state sequences (see Methods).

While the two TC methods sometimes had lower probability of finding either false UP or false DOWN states compared to the EDHMM method, they did not provide adequate protection against both types of errors (figure 8B). Furthermore, the TC methods were much more likely to detect additional LFP states than the EDHMM method, and were also more likely to miss MP states (figure 8C). To quantitatively compare the performance of different algorithms we computed the percent change in *e_i_* and *e_s_* relative to the EDHMM method (figures 8D,E). Both the SMM-TC and Np-TC methods had significantly larger *e_i_* relative to the EDHMM method (SMM-TC: +19%, +5.7–37%; *p* = 0.020, two-sided Wilcoxon signed rank test, n = 9; Np-TC: +18%, 9.8–39%; *p* = 0.012; figure 8D). The increases in *e_s_* for the TC algorithms relative to the EDHMM were even larger (SMM-TC: +75%, +51–85%; *p* = 3.9e-3; Np-TC: 72%, 30–96%; *p* = 0.011; figure 8E). Thus, the EDHMM algorithm produced significantly improved agreement between state sequences classified in the MP and LFP, both in terms of the instantaneous error *e_i_* and the state error *e_s_*.

We also sought to evaluate the contribution of two key components of our method to the decoding accuracy: the explicit state duration models, and the variable state-conditional means. Thus, we compared the values of *e_i_* and *e_s_* obtained using an HMM (no explicit state duration models), and a ‘fixed-mean EDHMM” (where the state-conditional means were constrained to be constant), with those obtained using the full EDHMM. While using the simpler HMM did not result in substantially higher *e_i_* (+0.37%, −0.63–0.62%; *p* = 0.91), it did significant increase *e_s_* (+14%, 9.2–18%; *p* = 7.8e-3). Somewhat surprisingly, we found that constraining the state means to be constant did not produced a significant increase in either *e_i_* (+0.70%, −4.3–3.5%; *p* = 0.82) or *e_s_* (+4.4%, −2.9–21%; *p* = 0.20) compared to the full EDHMM. Consistent with the signal feature separability analysis, classifying LFP UDS from the HF-power rather than LF-amplitude produced significant increases in both *e_i_* (+55%, 38–174%; *p* = 3.9e-3) and *e_s_* (+114%, 49–197%; *p* = 3.9e-3).

## Discussion

We conclude by summarizing several of the key improvements provided by our UDS inference procedure. Firstly, the framework presented here can be used to infer UDS from different types of continuous signals (such as MPs, LFPs, and EEGs), so that one does not need to resort to different procedures when using different types of signals. Our method also allows for UDS inference from combinations of simultaneously recorded signals, so that multi-electrode recordings could be used to analyze global as well as region-specific UDS. Different signal features, or combinations of features (e.g. HF-power and LF-amplitude) can be used as desired in each case. Importantly, regardless of the exact experimental conditions or type of signal(s) used, the signal feature separability provides a natural criterion for selecting a set of signals and/or signal features, without the need to perform calibration experiments of any kind [26]. When combining information from multiple signals and/or signal features, an important strength of a probabilistic generative model such as the EDHMM is that it will automatically account for correlations between the signal components, as well as differences in the information each component provides, when inferring the state sequence. Indeed, we found that the separability of the LF-amplitude, and particularly the HF-power, was highly variable across recordings, suggesting a strong advantage for adaptive methods when considering multiple signal features.

The flexibility of the HMM framework presented here avoids the need for subjectively chosen rules or model parameters which are unlikely to be optimal for a broad range of experimental conditions. Our algorithm also effectively handles the non-stationarities present in large datasets by allowing the state-conditional means of the signal features to vary in time, as well as by allowing inference of model parameters across discontinuous segments of data interrupted by periods of desynchronized activity. Another important feature of the algorithm is that it naturally provides a direct measure of uncertainty for the inference results in terms of the posterior distribution of the latent state variables. This could allow for identification of state ‘transition regions’, as well as the selection of particular segments of data for analysis where the estimated uncertainty about the hidden state sequence is sufficiently low (e.g. clear individual UP and DOWN states, or data epochs with particularly well-defined UP and DOWN states).

We use simultaneous LFP and MP recordings to confirm these benefits, showing that our method produces significant improvements in two different measures of the agreement between LFP and MP UDS. These results suggest that the EDHMM method outperforms threshold-crossing type methods regardless of the procedure used to select the fixed threshold. It is worth noting that the strength of this “MP-LFP comparison” metric in quantifying the accuracy of a particular algorithm for inferring LFP UDS is limited by several factors, including the instantaneous variability of UDS across neurons, as well as any ambiguity in inferring UDS from the MP. In particular, previous work [25] has shown that the agreement of simultaneously recorded MP and LFP UDS can be greater than the agreement of the UDS of two simultaneously recorded MP signals. Such limitations could explain the variable nature of the improvements in MP-LFP agreement seen with the EDHMM method, and its apparent lack of improvement over the ‘fixed-mean’ EDHMM. Thus, in addition to this quantitative measure of the accuracy of LFP UDS inference, we also emphasize other qualitative strengths of the methods presented here. Finally, we note that our EDHMM method could also be extended to include both continuous and point-process observations to allow UDS inference based on extracellular spikes as well as LFP signals, as done in [26] using a threshold-crossing type algorithm, and as suggested by [21] within the EDHMM framework.
